# HIV Preexposure Prophylaxis Utilization and Reasons for Never Using Preexposure Prophylaxis Among Transfeminine Persons in the United States: Findings From the Transgender Women’s Internet Survey and Testing (TWIST) Study

**DOI:** 10.1093/ofid/ofag073

**Published:** 2026-03-09

**Authors:** Duygu Islek, Travis Sanchez, Stefan Baral, Joanna A Caldwell, Jennifer L Glick, Irah Lucas, Supriya Sarkar, Leigh Ragone, Annemiek de Ruiter, Mariah Valentine-Graves, Savannah Winter, Vani Vannappagari

**Affiliations:** Rollins School of Public Health, Emory University, Atlanta, Georgia, USA; Rollins School of Public Health, Emory University, Atlanta, Georgia, USA; Johns Hopkins School of Public Health, Johns Hopkins University, Baltimore, Maryland, USA; Rollins School of Public Health, Emory University, Atlanta, Georgia, USA; Community Health Science and Policy, Health Sciences Center, Louisiana State University, New Orleans, Louisiana, USA; Rollins School of Public Health, Emory University, Atlanta, Georgia, USA; ViiV Healthcare, Durham, North Carolina, USA; ViiV Healthcare, Durham, North Carolina, USA; ViiV Healthcare, London, United Kingdom; Rollins School of Public Health, Emory University, Atlanta, Georgia, USA; Rollins School of Public Health, Emory University, Atlanta, Georgia, USA; ViiV Healthcare, Durham, North Carolina, USA

**Keywords:** HIV, preexposure prophylaxis, PrEP, transfeminine

## Abstract

**Background:**

Transfeminine persons in the United States face a high burden of human immunodeficiency virus (HIV), yet national data on preexposure prophylaxis (PrEP) use remain limited. We examined PrEP utilization, adherence, and persistence and reasons for never using PrEP among a national sample of transfeminine persons.

**Methods:**

Sexually active transfeminine persons aged ≥15 years without HIV were recruited online through the Transgender Women's Internet Survey and Testing (TWIST) Study, a national cross-sectional survey conducted between June 2023 and October 2024. Multivariable Poisson regression was used to estimate adjusted prevalence ratios for characteristics associated with current PrEP use. Reasons for never using PrEP were examined descriptively by age group.

**Results:**

Among 1656 participants, 6% were currently using PrEP and 86% had never used PrEP. Among current users (n = 96), 94% used oral PrEP and 6% used long-acting injectable (LA) PrEP. Among the 32 participants who reported using <30 daily PrEP doses in the past 30 days, 25% indicated that they were using event-driven (on-demand) PrEP, taking it only when they anticipated having sex. In multivariable models, current PrEP use was higher among participants aged ≥40 years, Black participants, and participants reporting a sexually transmitted infection diagnosis, multiple sexual partners, illicit drug use, or prescribed medication use. Common reasons for never using PrEP among participants aged 15–24 years included insurance-related privacy and disclosure concerns and transportation barriers, while among participants aged ≥25 years, reasons included loss of insurance, side-effect concerns, and monogamous partnerships.

**Conclusions:**

PrEP uptake among transfeminine persons remains low, with distinct age-specific barriers. Tailored interventions are needed. LA PrEP may help address challenges related to adherence and disclosure, particularly among younger individuals.

## Teaser

In a national survey of transfeminine persons without HIV, preexposure prophylaxis (PrEP) use was low (6%), with age-specific barriers including insurance concerns, transportation, and side-effect worries. Long-acting injectable PrEP may improve uptake and adherence.

Transfeminine persons in the United States (US) continue to experience a disproportionately high burden of human immunodeficiency virus (HIV). A recent study reported an overall HIV incidence of 5.5 per 1000 person-years among transgender women residing in the eastern and southern regions of the country [1]. Broader national surveillance data underscore the severity of this burden, with 40% of transgender women sampled across 7 major US cities found to be living with HIV [2]. Studies have shown that preexposure prophylaxis (PrEP) is an efficacious approach for HIV prevention among transfeminine individuals, who are disproportionately burdened by the risk of HIV acquisition [3]. However, information about PrEP utilization among transfeminine individuals remains limited with no nationwide data available [4, 5]. Existing studies have been limited by small sample sizes, geographically restricted cohorts, or inconsistent measurement of PrEP use, leaving critical gaps in understanding PrEP utilization across the US.

Transfeminine persons may face various challenges that could lead to either not initiating PrEP or discontinuing its use at various stages of the PrEP care continuum [6, 7]. Several factors have been proposed to explain the low uptake of PrEP among transfeminine persons, including concerns of potential interactions between gender-affirming hormone therapy and PrEP medications [8], as well as limited access to healthcare providers who offer culturally sensitive and clinically appropriate care [4]. Prior research has identified significant disparities in PrEP care engagement among transfeminine persons, with differences observed across age, race, geographic region, sexual behavior, history of sexually transmitted infections (STIs), and substance use [2, 9]. Despite these findings, important gaps remain in understanding how these factors influence PrEP use patterns among transfeminine persons nationally. Even among transfeminine individuals who initiate PrEP, adherence to effective dosing protocols and sustained long-term use remain suboptimal [6].

Transfeminine individuals face distinct barriers that must be accounted for when designing tailored PrEP interventions [10]. However, our understanding of the sociobehavioral characteristics of transfeminine persons across the PrEP care continuum is limited [7]. Data on the underlying reasons for never initiating or prematurely discontinuing PrEP remain especially scarce [11, 12]. These motivations may vary substantially by age, with younger individuals potentially facing different challenges and concerns compared to older age groups [10]. Clarifying these age-specific and behavioral factors is essential for informing targeted, culturally responsive PrEP strategies that can improve both uptake and persistence. In response, we aimed to describe the sociodemographic and behavioral characteristics of transfeminine persons by PrEP use, adherence, and persistence and examine reasons for never initiating PrEP among transfeminine persons in the US.

## METHODS

### Study Design and Analytic Sample

The TWIST (Transgender Women’s Internet Survey and Testing) Study is an online cross-sectional survey that collects extensive information on transfeminine individuals living in the US [13, 14]. The data collected include sociodemographic characteristics, sexual behaviors, HIV and STI testing and diagnosis, and involvement with HIV prevention services and substance use among transfeminine persons. Transfeminine persons were recruited using a targeted online sampling strategy to reach transfeminine persons across diverse geographic and sociodemographic contexts in the US. Participants were recruited using targeted ads placed on websites, social media platforms, and mobile apps frequently used by transfeminine people. Recruitment was monitored and adjusted to enhance heterogeneity by age, race/ethnicity, region, and urbanicity. This strategy enabled efficient recruitment of a large, diverse national sample. Individuals who clicked on an advertisement or accessed the survey through an email link were directed to a screening questionnaire to determine their eligibility. To reduce duplicate or fraudulent entries, we implemented a 2-stage validation process combining automated and manual review. Automated checks flagged responses based on failed reCAPTCHA verification, implausibly short completion times, incomplete surveys, missing recruitment-source identifiers, mismatched demographic items, or completion of a hidden bot-detection question. As part of duplicate-response prevention, internet protocol (IP) addresses were temporarily collected to identify repeated submissions and were removed and replaced with study IDs prior to analysis. Submissions passing automated checks underwent manual review, including assessment of IP and email patterns. Responses with multiple flags or inconsistencies were evaluated case by case, and only validated submissions were retained.

Eligibility criteria were (1) age ≥15 years; (2) assigned male sex at birth; (3) currently identifying gender as female, transgender woman, or transfeminine nonbinary; (4) ever engaged in oral, anal, or vaginal sex; and (5) currently residing within the US. Eligible individuals were guided to an online consent form, followed by the complete web-based survey. After finishing the survey, respondents received a $10 digital gift card as compensation. The TWIST Study's methodology has been previously described in detail [13].

The TWIST study recruited participants and collected survey data continuously from June 2023 through October 2024; no data outside this interval were included. Statistical analyses were performed between December 2024 and March 2025. The final analytic sample was determined by applying additional selection criteria: Participants were required to have engaged in oral, anal, or vaginal intercourse within the preceding 12-month period and to have no self-reported HIV diagnosis. The research protocol was approved by the Institutional Review Board (IRB) of Emory University (IRB number: IRB00108784). The Emory University IRB approved a waiver of parental/guardian permission for participants aged 15–17 years, as requiring parental consent could increase risk through involuntary disclosure of gender identity or sexual behavior. The study was classified as minimal risk, and all minor participants provided informed assent.

### Outcome Measures

PrEP-related outcomes were evaluated descriptively and included 4 primary domains [15]: PrEP use status, PrEP adherence and persistence, reasons for PrEP discontinuation, and reasons for never PrEP use.

PrEP use status was categorized as (1) never PrEP use; (2) ever used PrEP but not in the past 12 months; and (3) used PrEP within the past 12 months, including both current users and those who recently discontinued use.

PrEP adherence [16] and persistence [2] were described among participants currently using PrEP and were evaluated separately for oral and long-acting injectable (LA) PrEP [17]. Among participants using daily oral PrEP, adherence was operationalized based on self-reported number of doses taken in the past 30 days (≤15 doses, 16–29 doses, or 30 doses). Participants reporting <30 doses were additionally asked about their intended dosing strategy (daily vs only when anticipating sex). PrEP persistence among oral PrEP users was defined as the self-reported duration of consecutive daily PrEP use in months. If participants used PrEP for <12 months, they were asked if they took any other PrEP medication in the past 12 months and the reason for switching.

Among participants using LA PrEP, adherence was assessed using self-reported number of injections received and number of injections missed in the past 12 months. LA PrEP users were additionally asked where they got their LA PrEP injections (private doctor's office, sexual health clinic, health center, pharmacy, at home, or other location). Daily oral and LA PrEP outcomes were evaluated separately.

PrEP discontinuation was assessed among participants who stopped using PrEP within the past 12 months or earlier and who were not current users. These participants were asked to report reasons for discontinuation from a predefined list of options.

Reasons for never PrEP use were collected from participants who reported never having used PrEP, using a predefined list of potential reasons. Reasons for PrEP discontinuation and for never PrEP use were examined descriptively overall, and reasons for never PrEP use were additionally stratified by age group (15–24 years vs ≥25 years).

Event-driven (on-demand) PrEP was not evaluated as a primary analytic outcome. Instead, participants using daily oral PrEP who reported taking <30 doses in the past 30 days were asked about their intended dosing strategy; those indicating use “only when I have sex” were descriptively classified as using event-driven PrEP. The survey questions used to assess and describe PrEP-related outcomes are detailed in Supplementary Table 1.

### Covariate Measures

Sociodemographic covariates included age (categorized into 15–24, 25–29, 30–39, and ≥40 years), race/ethnicity (non-Hispanic White, Hispanic/Latinx, non-Hispanic Black, and other or multiple races), health insurance type (private only, public only, none, or other/multiple), urbanicity of county of residence (classified using the 2013 National Center for Health Statistics Urban–Rural Classification Scheme [18] based on self-reported ZIP [postal] code), and US census region.

Behavioral covariates [14] reflected self-reported experiences within the past 12 months. Condomless anal sex and condomless vaginal sex were defined as reporting at least 1 episode of anal or vaginal intercourse without a condom, respectively. Number of sexual partners was categorized as 1 versus >1 partner. Self-reported STI diagnosis captured whether participants reported having been diagnosed with any STI by a healthcare provider during the prior 12 months.

Substance use variables included self-reported marijuana use and illicit drug use other than marijuana (eg, stimulants, opioids, or other recreational drugs) in the past 12 months, each categorized as yes or no. Exchange sex was defined as reporting having exchanged money or drugs for sex during the previous 12 months [2].

Participants were additionally asked about healthcare engagement–related behaviors, including whether they were currently taking any daily prescription medications and whether they had received any prescribed injections (excluding vaccines) in the past 12 months. For injection use, participants reported whether injections were self-administered, administered by someone else, or both.

The uptake of gender affirmation care [19] was determined by inquiring whether participants had used hormones or hormone blockers for medical transition or gender affirmation within the last 12 months.

### Statistical Analysis

We tabulated and descriptively summarized the frequency distribution of the participant characteristics in the whole sample. If participants were currently using oral PrEP, we described the frequency of PrEP adherence, intention to take PrEP, PrEP persistence, and switching. If participants were LA PrEP users, we described the distribution of the location where they got their LA PrEP injections, number of injections they received in the past 12 months, and number of injections they missed in the past 12 months.

We conducted bivariate analyses to compare sociodemographic and behavioral characteristics across 3 PrEP use categories: (1) never used PrEP, (2) ever used PrEP but not in the past 12 months, and (3) used PrEP in the past 12 months. For all categorical variables, we used χ^2^ tests (or Fisher exact tests when expected cell counts were <5) to assess whether distributions differed across PrEP use categories. Statistical significance was defined as 2-sided *P* < .05.

We used Poisson regression to examine the association between participant characteristics and current PrEP use. We included all sociobehavioral characteristics in our adjusted models. We report unadjusted and adjusted prevalence ratios and 95% confidence intervals.

We examined the distribution of reasons for PrEP discontinuation. Finally, we examined the distribution of reasons for never using PrEP in the whole sample and stratified by age group (15–24 years and ≥25 years). The data analysis was conducted with software package SAS version 9.4 (SAS Institute, Cary, North Carolina, USA).

## RESULTS

Of 1656 participants, 93% were <40 years old, 75% were Non-Hispanic White, and 11% were Hispanic/Latinx (Table 1). The majority of participants had private health coverage, 41% resided in large central metropolitan counties, and two-thirds of the participants resided in the southern or western US. Considering behavioral characteristics, approximately 40% of participants engaged in condomless anal or vaginal sex and about half reported >1 sexual partner within the past 12 months. More than 50% of the individuals reported using marijuana in the past 12 months and 81% of participants were taking daily prescription medications. Additionally, 76% reported using hormones for gender affirmation (Table 1). Overall, 6% (103/1656) of participants were currently using PrEP, 3% (44/1656) of participants were not current users but used PrEP in the past 12 months, 4% (61/1656) reported that they ever used PrEP but not in the past 12 months, and 86% (1417/1656) never used PrEP.

**Table 1.** Characteristics of US Participants in the Transgender Women's Internet Survey and Testing (TWIST) Study, 2023–2024 (N = 1656)

Characteristic	No. (%)
Age, y	
15–24	848 (51.2)
25–29	365 (22.0)
30–39	330 (19.9)
≥40	113 (6.8)
Race/Ethnicity	
Black, non-Hispanic	15 (0.9)
Hispanic or Latinx	185 (11.2)
White, non-Hispanic	1236 (75.1)
Other or multiple races	210 (12.8)
Health insurance	
None	133 (8.2)
Private only	1162 (71.2)
Public only	228 (14.0)
Other/multiple	108 (6.6)
Urbanicity of county of residence	
Large central metropolitan	686 (41.5)
Large fringe metropolitan	372 (22.5)
Medium metropolitan	334 (20.2)
Small metropolitan, micropolitan, and non-core	261 (15.8)
Census region	
Northeast	288 (17.4)
Midwest	336 (20.3)
South	477 (28.8)
West	554 (33.5)
STI diagnosis in past 12 mo	
No	1611 (97.3)
Yes	45 (2.7)
Condomless anal sex in past 12 mo	
No	981 (59.2)
Yes	675 (40.8)
Condomless vaginal sex in past 12 mo	
No	931 (56.3)
Yes	723 (43.7)
Number of sex partners in past 12 mo	
1	827 (50.4)
>1	815 (49.6)
Marijuana use in past 12 mo	
No	733 (44.3)
Yes	923 (55.7)
Other illicit drug use in past 12 mo	
No	1096 (66.2)
Yes	560 (33.8)
Currently taking daily prescription pills	
No	304 (18.5)
Yes	1342 (81.5)
Injection of prescribed medication in past 12 mo	
No	1042 (63.5)
Yes, injected myself	395 (24.1)
Yes, someone else injected	120 (7.3)
Yes, injected by both myself and someone else	83 (5.1)
Exchange sex in past 12 mo	
No	1540 (93.0)
Yes	116 (7.0)
Hormone use for gender affirmation in past 12 mo	
No	397 (23.9)
Yes	1258 (76.0)
Currently using HIV PrEP	
No	1553 (93.8)
Yes	103 (6.2)
Used HIV PrEP in past 12 mo, but discontinued	
No	1612 (97.3)
Yes	44 (2.7)
Ever used HIV PrEP but not in the past 12 mo	
No	1595 (96.3)
Yes	61 (3.7)
Never used HIV PrEP	
No	239 (14.4)
Yes	1417 (85.6)

Abbreviations: HIV, human immunodeficiency virus; PrEP, preexposure prophylaxis; STI, sexually transmitted infection.

Among current users, 61% (59/96) were using emtricitabine/tenofovir disoproxil fumarate, 32% (31/96) were using emtricitabine/tenofovir alafenamide, and 6% (6/96) were using LA PrEP (cabotegravir). Among current oral PrEP users, 48% (43/90) had been using PrEP for >12 months. Among participants who have been using oral PrEP for 12 months or less, 6% (3/47) had switched to another oral PrEP medication and all of them (3/3) reported their reason for switching as concern about side effects. Among daily oral PrEP users, 64% (58/90) reported having used 30 doses of daily oral PrEP in the last 30 days whereas 18% (16/90) reported that they took 16–29 doses and 18% (16/90) reported that they took ≤15 doses in the last 30 days. Among participants who took <30 doses (n = 32), 72% (23/32) reported that they intended to take oral PrEP daily but did not, and 25% (8/32) reported that they intended to take PrEP only when they had sex. Among 6 LA PrEP users (n = 6), 4 participants took their injections in a sexual health clinic, 1 participant in a private doctor's office, and 1 participant in a health center. Considering adherence to LA PrEP, 4 of 5 LA PrEP users reported that they had not missed any injections while 1 participant reported missing 2 injections in the last 12 months.

Table 2 presents bivariate comparisons of sociodemographic and behavioral characteristics across PrEP use history. PrEP use history differed significantly by age group (*P* < .001), with a higher proportion of recent PrEP use observed among older participants, particularly those aged 30–39 years and ≥40 years, compared with younger participants aged 15–24 years. PrEP use also varied significantly by health insurance status (*P* = .0006) and urbanicity of county of residence (*P* < .001). Participants with public insurance and those residing in large central metropolitan areas were more likely to report PrEP use in the past 12 months than those with private insurance or living in less urbanized areas. In bivariate comparisons, participants reporting condomless anal sex (*P* < .001), multiple sexual partners (*P* < .001), marijuana use (*P* = .0079), or use of other illicit drugs (*P* < .001) in the past 12 months were more likely to report recent PrEP use compared with participants who did not report these behavioral characteristics (Table 2).

**Table 2.** Characteristics of Participants Who Never Used Preexposure Prophylaxis (PrEP), Ever Used PrEP but Stopped >12 Months Ago, and Who Have Been Using PrEP in the Past 12 Months, Transgender Women’s Internet Survey and Testing (TWIST) Study, 2023–2024

Characteristic	Never Used PrEP (n = 1417)	Ever Use Before 12 Months (n = 61)	Used in the Last 12 Months^a^ (n = 147)	*P* Value^b^
Age, y				
15–24	770 (92.4)	16 (1.9)	47 (5.6)	<.001
25–29	301 (84.1)	20 (5.6)	37 (10.3)	
30–39	258 (79.4)	21 (6.5)	46 (14.2)	
≥40	88 (80.7)	4 (3.7)	17 (15.6)	
Race/Ethnicity				
Black, non-Hispanic	12 (80.0)	0 (0.0)	3 (20.0)	.4180
Hispanic or Latinx	153 (85.5)	6 (3.4)	20 (11.2)	
White, non-Hispanic	1069 (87.9)	47 (3.9)	100 (8.2)	
Other or multiple races	175 (85.0)	8 (3.9)	23 (11.2)	
Health insurance				
None	114 (88.4)	4 (3.1)	11 (8.5)	.0006
Private only	1010 (88.2)	37 (3.2)	98 (8.6)	
Public only	175 (77.8)	17 (7.6)	33 (14.7)	
Other/multiple	97 (93.3)	3 (2.9)	4 (3.8)	
Urbanicity of county of residence				
Large central metro	545 (81.1)	35 (5.2)	92 (13.7)	<.001
Large fringe metro	328 (90.1)	8 (2.2)	28 (7.7)	
Medium metro	297 (90.3)	13 (4.0)	19 (5.8)	
Small metro, micropolitan, and non-core	244 (94.9)	5 (1.9)	8 (3.1)	
Census region				
Northeast	240 (84.8)	7 (2.5)	36 (12.7)	.1093
Midwest	287 (87.5)	16 (4.9)	25 (7.6)	
South	422 (89.4)	17 (3.6)	33 (7.0)	
West	467 (86.3)	21 (3.9)	53 (9.8)	
STI diagnosis in past 12 mo				
No	1395 (88.3)	59 (3.7)	126 (8.0)	
Yes	22 (48.9)	2 (4.4)	21 (46.7)	
Condomless anal sex in past 12 mo				
No	878 (91.4)	26 (2.7)	57 (5.9)	<.001
Yes	539 (81.2)	35 (5.3)	90 (13.6)	
Condomless vaginal sex in past 12 mo				
No	782 (85.7)	38 (4.2)	92 (10.1)	.1453
Yes	633 (89.0)	23 (3.2)	55 (7.7)	
Number of sex partners in past 12 mo				
1	774 (95.3)	22 (2.7)	16 (2.0)	<.001
>1	634 (79.2)	39 (4.9)	128 (16.0)	
Marijuana use in past 12 mo				
No	642 (89.5)	28 (3.9)	47 (6.6)	.0079
Yes	775 (85.4)	33 (3.6)	100 (11.0)	
Other illicit drug use in past 12 mo				
No	988 (92.0)	30 (2.8)	56 (5.2)	<.001
Yes	429 (77.9)	31 (5.6)	91 (16.5)	
Currently taking daily prescription pills				
No	284 (94.7)	9 (3.0)	7 (2.3)	<.001
Yes	1123 (85.4)	52 (4.0)	140 (10.6)	
Injection of prescribed medication in past 12 mo				
No	946 (92.7)	24 (2.4)	51 (5.0)	<.0001
Yes, injected myself	304 (77.2)	29 (7.4)	61 (15.5)	
Yes, someone else injected	97 (82.9)	4 (3.4)	16 (13.7)	
Yes, injected by both myself and someone else	58 (72.5)	4 (5.0)	18 (22.5)	
Exchange sex in past 12 mo				<.0001
No	1336 (88.4)	51 (3.4)	124 (8.2)	
Yes	81 (71.1)	10 (8.8)	23 (20.2)	
Hormone use for gender affirmation in past 12 mo				<.0001
No	368 (95.6)	5 (1.3)	12 (3.1)	
Yes	1048 (84.6)	56 (4.5)	135 (10.9)	

Data are presented as No. (%) unless otherwise indicated.

Abbreviations: PrEP, preexposure prophylaxis; STI, sexually transmitted infection.

^a^Includes participants who were currently using PrEP (n = 103).

^b^ *P* values reflect bivariate comparisons across the 3 PrEP use categories. The χ^2^ test was used unless expected cell counts were <5, in which case Fisher exact test was applied. Two-sided *P* < .05 was considered statistically significant.

In multivariable modeling, current PrEP use was higher among Black participants, among participants who reported having an STI diagnosis, >1 partner, illicit drug use (other than marijuana), or a prescribed medication injection in the past 12 months, and among participants who were concurrently taking daily prescription pills (Table 3). On the other hand, PrEP use was lower among participants aged 15–29 years compared to participants aged ≥40 years (Table 3).

**Table 3.** Participant Characteristics by Current HIV Preexposure Prophylaxis Use, Transgender Women’s Internet Survey and Testing (TWIST) Study, 2023–2024

Characteristic	Currently Using PrEP (n = 103)	Not Using PrEP (n = 1550)	PR (95% CI)	Adjusted PR (95% CI)
Age, y				
15–24	33 (3.9)	815 (96.1)	0.34 (.18–.62)	**0.44** (**.24–.82)**
25–29	21 (5.8)	343 (94.2)	0.50 (.26–.97)	**0.42** (**.22–.80)**
30–39	36 (11.0)	292 (89.0)	0.95 (.53–1.73)	0.87 (.48–1.59)
≥40	13 (11.5)	100 (88.5)	ref	ref
Race/Ethnicity				
Black, non-Hispanic	3 (20.0)	12 (80.0)	3.63 (1.29–10.25)	**4.32** (**1.48–12.63)**
Hispanic or Latinx	13 (7.0)	172 (93.0)	1.28 (.72–2.26)	1.01 (.56–1.85)
White, non-Hispanic	68 (5.5)	1166 (94.5)	ref	ref
Other or multiple races	18 (8.6)	191 (91.4)	1.56 (.95–2.57)	1.49 (.94–2.36)
Health insurance				
None	8 (6.0)	125 (94.0)	1.07 (.53–2.19)	0.79 (.40–1.54)
Private only	65 (5.6)	1096 (94.4)	ref	ref
Public only	28 (12.4)	198 (87.6)	2.21 (1.45–3.37)	1.55 (.98–2.45)
Other/multiple	1 (0.9)	107 (99.1)	0.17 (.02–1.18)	0.17 (.02–1.27)
Urbanicity of county of residence				
Large central metro	63 (9.2)	622 (90.8)	ref	ref
Large fringe metro	21 (5.7)	350 (94.3)	0.62 (.38–.99)	1.06 (.65–1.73)
Medium metro	15 (4.5)	319 (95.5)	0.49 (.28–.84)	0.81 (.45–1.43)
Small metro, micropolitan, and non-core	4 (1.5)	257 (98.5)	0.17 (.06–.45)	0.25 (.08–.77)
Census region				
Northeast	26 (9.0)	262 (91.0)	ref	ref
Midwest	19 (5.7)	316 (94.3)	0.63 (.36–1.11)	0.81 (.49–1.36)
South	21 (4.4)	456 (95.6)	0.49 (.28–.85)	0.76 (.44–1.33)
West	37 (6.7)	516 (93.3)	0.74 (.46–1.20)	0.76 (.49–1.19)
STI diagnosis in past 12 mo				
No	88 (5.5)	1521 (94.5)	ref	ref
Yes	15 (34.1)	29 (65.9)	6.23 (3.94–9.86)	**2.97** (**1.73–5.09)**
Condomless anal sex in in past 12 mo				
No	45 (4.6)	933 (95.4)	ref	ref
Yes	58 (8.6)	617 (91.4)	1.87 (1.28–2.72)	1.14 (.77–1.69)
Condomless vaginal sex in past 12 mo				
No	62 (6.7)	869 (93.3)	ref	ref
Yes	41 (5.7)	679 (94.3)	0.86 (.58–1.25)	0.77 (.54–1.11)
Number of sex partners in past 12 mo				
1	13 (1.6)	813 (98.4)	ref	ref
>1	89 (10.9)	725 (89.1)	6.95 (3.91–12.33)	**4.18** (**2.29–7.63)**
Marijuana use in past 12 mo				
No	33 (4.5)	698 (95.5)	ref	ref
Yes	70 (7.6)	852 (92.4)	1.68 (1.12–2.51)	0.81 (.47–1.40)
Other illicit drug use in past 12 mo				
No	38 (3.5)	1056 (96.5)	ref	ref
Yes	65 (11.6)	494 (88.4)	3.35 (2.27–4.93)	**1.94** (**1.13–3.31)**
Currently taking daily prescription pills				
No	3 (1.0)	301 (99.0)	ref	ref
Yes	100 (7.5)	1239 (92.5)	7.57 (2.42–23.70)	**4.71** (**1.33–16.62)**
Injection of prescribed medication in past 12 mo				
No	35 (3.4)	1005 (96.6)	ref	ref
Yes, injected myself	44 (11.2)	350 (88.8)	3.32 (2.16–5.09)	**1.64** (**1.02–2.62)**
Yes, someone else injected	11 (9.2)	109 (90.8)	2.72 (1.42–5.22)	1.60 (.85–3.02)
Yes, injected by both myself and someone else	12 (14.5)	71 (85.5)	4.30 (2.32–7.96)	**2.14** (**1.13–4.05)**
Exchange sex in past 12 mo				
No	86 (5.6)	1451 (94.4)	ref	Ref
Yes	17 (14.7)	99 (85.3)	2.62 (1.61–4.25)	1.37 (.85–2.21)
Hormone use for gender affirmation in past 12 mo				
No	10 (2.5)	387 (97.5)	ref	Ref
Yes	93 (7.4)	1162 (92.6)	3.06 (1.56–6.01)	0.77 (.38–1.55)

Data are presented as No. (%) unless otherwise indicated. Bold text indicates statistical significance.

Abbreviations: CI, confidence interval; HIV, human immunodeficiency virus; PrEP, preexposure prophylaxis; PR, prevalence ratio; STI, sexually transmitted infection.

**Figure 1.**
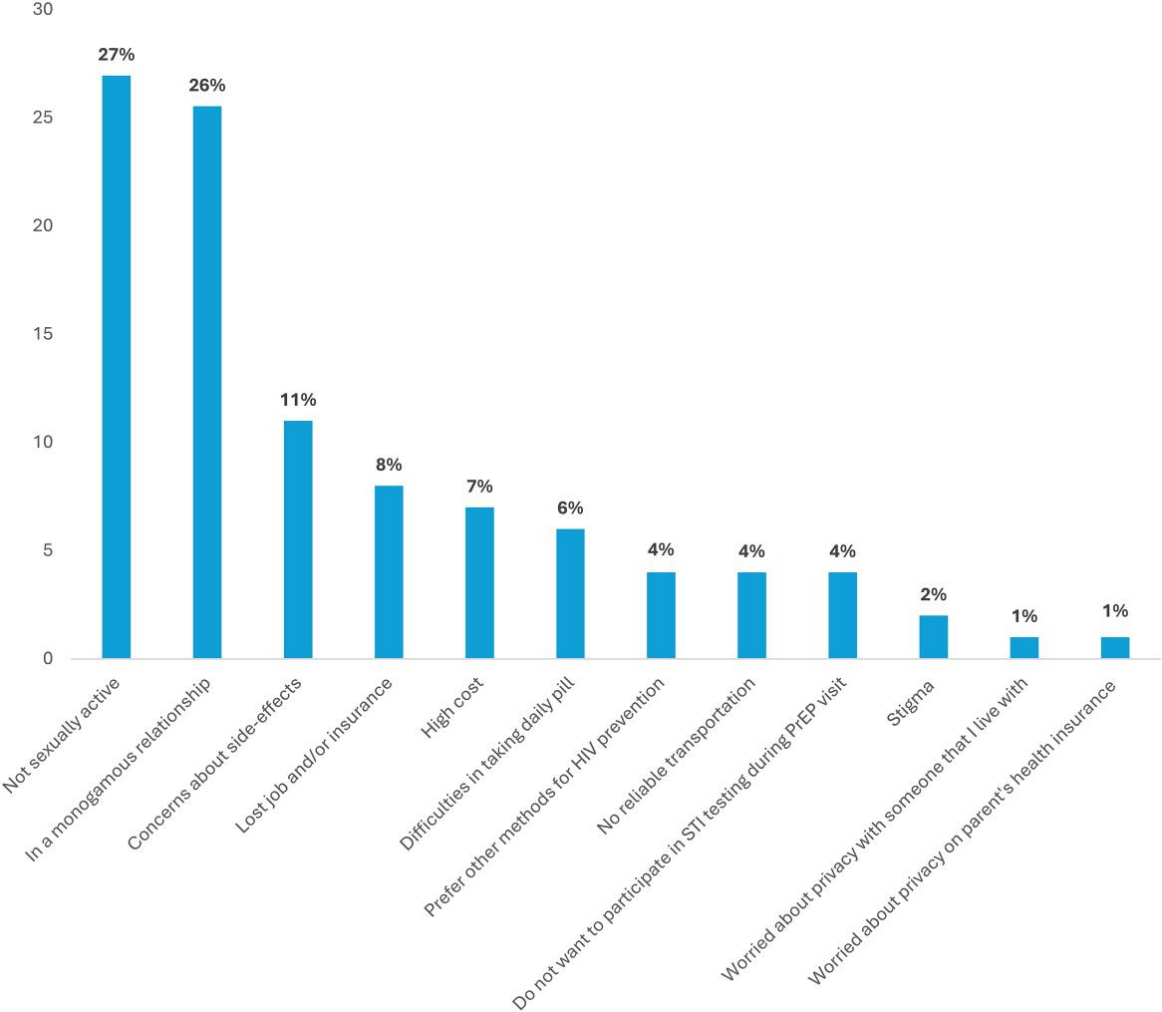
Reasons for PrEP discontinuation among past PrEP user transfeminine persons, the Transgender Women's Internet Survey and Testing (TWIST) Study, United States, 2023–2024 (n = 105). Past PrEP users are 105 participants who used PrEP in the last 12 months or earlier. Abbreviations: HIV, human immunodeficiency virus; PrEP, preexposure prophylaxis; STI, sexually transmitted infection.

The most common reasons for PrEP discontinuation were being not sexually active (27%) and being in a monogamous relationship (26%). Other notable reasons were concerns about side effects (11%) and loss of job or insurance (8%) (Figure 1).

**Figure 2.**
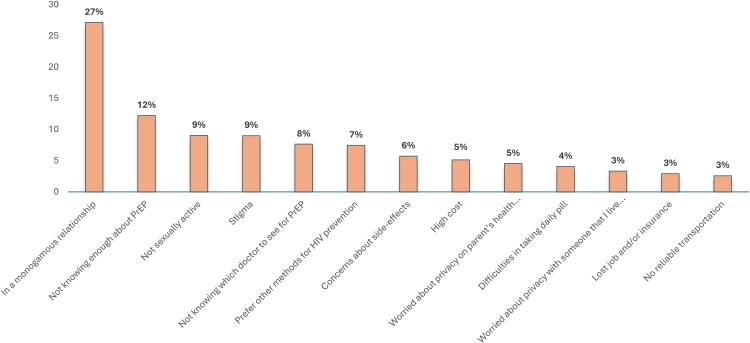
Reasons for never PrEP use among transfeminine persons, the Transgender Women's Internet Survey and Testing (TWIST) Study, United States, 2023–2024 (n = 1417). Participants were allowed to choose >1 option. Abbreviations: HIV, human immunodeficiency virus; PrEP, preexposure prophylaxis.

Among all participants who never used PrEP (n = 1417), the most common reasons for never using PrEP were being in a monogamous relationship (27%), not knowing enough about PrEP to make a decision (12%), being not sexually active (9%), and healthcare-related or PrEP-related stigma (9%) (Figure 2).

**Figure 3.**
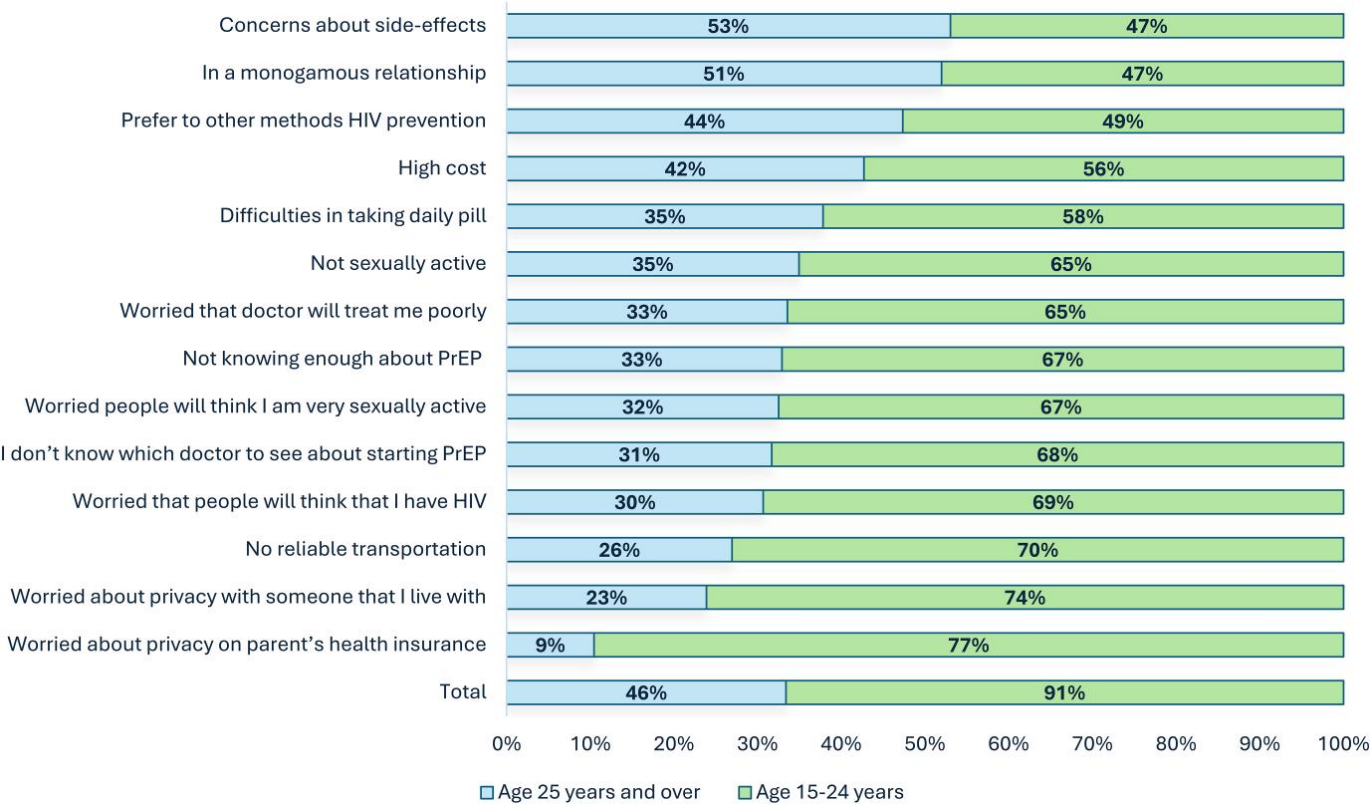
Reasons for never using PrEP among US transfeminine persons, stratified by age group, the Transgender Women's Internet Survey and Testing (TWIST) Study, United States, 2023–2024 (n = 1417). Participants aged 15–24 years, n = 770; participants aged ≥25 years, n = 647. Participants were allowed to choose >1 option. Abbreviations: HIV, human immunodeficiency virus; PrEP, preexposure prophylaxis.

In the stratified analysis, among participants aged 15–24 years (n = 770), common reasons for never using PrEP included concerns about privacy due to being on a parent's health insurance, worries about privacy with someone they live with, and lack of reliable transportation (Figure 3). These reasons were notably less common among participants aged ≥25 years (n = 647). Conversely, among participants aged ≥25 years, more common reasons for not initiating PrEP included losing a job or insurance coverage, concerns about potential side effects, and being in a monogamous relationship.

## DISCUSSION

Despite over a decade of availability, PrEP use was low within our nationwide, online sample of transfeminine persons, with only 6% of participants reporting current use. This underutilization persists even though PrEP is a well-established and effective method of HIV prevention and transfeminine persons remain disproportionately affected by the HIV epidemic [1, 3]. The findings presented here include new insights into the sociodemographic and behavioral characteristics of PrEP use, adherence, and reasons for never use among transfeminine persons, with age-specific heterogeneities highlighting the need for tailored interventions. Beyond initiation, maintaining effective PrEP coverage remains a challenge, as many oral PrEP users in our study reported suboptimal adherence or use limited to sexual activity, which may not provide sufficient protection given the high rates of condomless sex, multiple partners, and recent STI diagnoses observed in this population. These findings underscore the need for strategies to support consistent PrEP use or alternative options like long-acting PrEP to ensure sustained HIV prevention among transfeminine individuals.

Transfeminine persons aged ≥25 years, Hispanic or Latinx participants, those with public insurance, and those living in large metropolitan areas were more likely to have used or be currently using PrEP. These results are consistent with prior studies [20, 21]. Importantly, under the US Preventive Services Task Force Grade A recommendation, both public and most private insurance plans are required to cover PrEP-related services without patient cost-sharing. As such, observed differences by insurance type likely reflect variation in access, implementation, and delivery of PrEP services rather than insurance coverage alone. Public insurance programs and safety-net health systems are often closely linked to community-based HIV prevention programs and may provide more proactive outreach, navigation support, and culturally competent care for transfeminine individuals. In contrast, barriers within some private healthcare settings, such as limited provider familiarity with PrEP for transfeminine patients or less consistent PrEP counseling, may still affect uptake despite mandated coverage. Additionally, access may vary by PrEP formulation: Generic oral PrEP is generally widely available, whereas LA PrEP may face additional barriers related to formulary inclusion, prior authorization, and clinic-based administration. Finally, higher PrEP use among participants residing in large metropolitan areas likely reflects the greater availability of urban HIV prevention infrastructure and specialized services uptake [20, 22].

Among younger participants (aged 15–24 years), reasons for never initiating PrEP were dominated by concerns related to insurance confidentiality and disclosure, particularly fear of parental insurance billing and inadvertent disclosure to household members. These barriers reflect structural vulnerabilities tied to dependence on family or shared living environments, where accessing PrEP may carry social or interpersonal risks beyond the individual [23–25]. Anticipated stigma and fear of judgment appeared especially pronounced in this age group, shaping avoidance of HIV prevention services even when perceived risk was present [23, 24, 26]. Notably, these concerns were among the least frequently reported barriers among older participants, underscoring a clear age-based divergence in PrEP access challenges. Together, these findings highlight the need for age-appropriate PrEP education, strengthened confidentiality protections, and youth-centered delivery models that reduce disclosure-related harms and enable younger transfeminine individuals to access HIV prevention services safely and autonomously.

A substantial proportion of participants reported limited knowledge about PrEP and anticipated stigma within healthcare settings as key reasons for never using PrEP. These findings point to persistent gaps in PrEP education at both the participant and provider levels. Insufficient counseling, limited provider familiarity with PrEP for transfeminine patients, and lack of culturally competent care may hinder informed decision-making and contribute to delayed or foregone PrEP initiation [27]. Improving provider training and integrating PrEP education into gender-affirming and routine healthcare settings remain critical to increasing PrEP uptake and persistence among transfeminine persons [28, 29].

Most common reasons for PrEP discontinuation were being not sexually active, being in a monogamous relationship, and concerns about side effects. Among participants who switched PrEP medications, all cited “side effects” as the motivating factor for switching. These findings are consistent with existing literature indicating that concerns about side effects, particularly potential drug–drug interactions with gender-affirming hormone therapy, can negatively impact PrEP persistence [30, 31]. Ensuring access to healthcare providers who can offer clear guidance on the safety of PrEP, including its concurrent use with hormone therapy, is essential to support continued use among transfeminine individuals.

Our findings also reinforce known associations between PrEP use and behavioral factors. PrEP use was more common among participants reporting multiple sexual partners and condomless sex in the past 12 months [2, 4]. Notably, nearly half of participants who reported an STI diagnosis in the past 12 months also reported current PrEP use. One possible explanation is the routine STI screening integrated into PrEP care, which may increase the detection of asymptomatic infections [32]. Supporting this, the majority of LA PrEP users in our study received their injections at STI clinics, where such screenings are readily available. Additionally, it may reflect a subgroup of individuals who are more proactive in seeking sexual health services overall, including both PrEP and STI testing. Importantly, this association should not be interpreted solely as evidence of behavioral disinhibition among PrEP users leading to higher STI risk. Instead, it underscores the complex relationship between PrEP use, health-seeking behaviors, and STI diagnoses, and highlights the need for continued sexual health education and prevention strategies alongside PrEP provision.

This study has limitations. We collected data on self-reported behaviors, which may introduce the possibility of recall and reporting biases [33]. Uptake of gender-affirming care was operationalized as self-reported use of gender-affirming hormones or puberty blockers in the past 12 months. While gender affirmation is a broader construct that also includes social, legal, and mental health dimensions, these domains were not comprehensively assessed in the TWIST survey and therefore could not be included. As such, this measure should be interpreted as a proxy for engagement in medical gender-affirming care and recent healthcare utilization, which is relevant to PrEP access, rather than a comprehensive measure of gender affirmation. Finally, our online recruitment strategy may affect the generalizability of findings; however, we employed targeted sampling approaches to enhance demographic diversity across age, geographic region, and race/ethnicity [34–36].

## CONCLUSIONS

Taken together, these data underscore the persistent gaps in PrEP uptake and retention among transfeminine persons in the US, particularly among younger individuals. Age-stratified analysis revealed unique barriers for PrEP initiation, with stigma, dependency, and concerns about disclosure disproportionately affecting younger participants. Addressing these issues will require age-specific, culturally competent interventions, improved integration of PrEP with gender-affirming care, and expanded access to LA PrEP. LA PrEP shows promise for overcoming adherence and privacy barriers, with prior research indicating high willingness to use it among transfeminine individuals [14, 37]. Overall, these strategies may help ensure that, in particular, young transfeminine persons receive equitable and sustained access to HIV prevention services.

## Supplementary Material

ofag073_Supplementary_Data

## Supplementary Data

Supplementary materials are available at *Open Forum Infectious Diseases* online. Consisting of data provided by the authors to benefit the reader, the posted materials are not copyedited and are the sole responsibility of the authors, so questions or comments should be addressed to the corresponding author.
