# Supplementary material for: HIV Preexposure Prophylaxis Utilization and Reasons for Never Using Preexposure Prophylaxis Among Transfeminine Persons in the United States: Findings From the Transgender Women’s Internet Survey and Testing (TWIST) Study
Source: Open Forum Infect Dis. 2026 Mar 9;13(3):ofag073. doi: 10.1093/ofid/ofag073 (PMC12970524; doi:10.1093/ofid/ofag073)
Supplement: ofag073_Supplementary_Data [file ofag073_supplementary_data.docx]

**S1 Table.** Questions used for describing PrEP outcomes

| Ever PrEP use | Have you ever taken PrEP? (Yes/No) |
| --- | --- |
| Current PrEP use | Are you currently taking PrEP? (yes/No) |
| Past PrEP use | In the past 12 months, have you taken PrEP? (yes/No) |
| Daily oral PrEP use adherence | In the last 30 days, about how many doses of PrEP did you take? |
| Daily oral PrEP use persistence | How many months in a row have you been taking PrEP? Less than 2 months / 2 to 6 months / 7 to 12 months/ 12 months or more |
| LA PrEP adherence & persistence | In the past 12 months, how many Apretude injections have you received?(1/2/3/4/5/6 or more) |
| LA PrEP adherence & persistence | In the past 12 months, how many Apretude injections did you miss? (open -ended question) |
| Reasons for r PrEP discontinuation | Which of the following describes the reason(s) why you stopped using PrEP the last time you were on it: (Check all that apply) |
| Reasons for never PrEP use | Which of the following describes the reason(s) why you have never taken PrEP? Choose all that apply. |
| Event-driven (on-demand PrEP use) | How frequently do you intend to take PrEP? (Daily/ only when I have sex/ some other schedule) |
